# The Privilege of Working From Home at the Time of Social Distancing

**DOI:** 10.1007/s10272-020-0891-3

**Published:** 2020-06-07

**Authors:** Armanda Cetrulo, Dario Guarascio, Maria Enrica Virgillito

**Affiliations:** 1 Institute of Economics, Sant’Anna - School of Advanced Studies Pisa, Piazza Martiri della Libertà, 33, 56127 Pisa, Italy; 2 Department of Economics and Law, Sapienza University of Rome, Via Del Castro Laurenziano 9, 00161 Rome, Italy

## Abstract

Although the common perception is that the pandemic is ‘the great equaliser’, workers’ tasks, contractual framework and position in the internal organisational hierarchy strongly affect their ability to work remotely.

The explosion of the COVID-19 pandemic and the ensuing policy of social distancing undertaken by many countries have put the organisation of production and of the work process under unprecedented stress.[1] Analyses of the pandemic's impact on the labour market are now spurring (Coibion et al., 2020) scary projections in terms of the number of jobs lost and related income losses. After just the first month, around 11 million European workers have experienced consequences related to the pandemic. An additional four million people are unemployed and an additional seven million short-term contract workers are at risk of losing their jobs, as estimated by the European Trade Union Confederation (2020). This combined supply and demand crisis recalls some massive policy interventions that date back to the Great Depression, namely reduced limits of deficit spending, direct bond purchasing by central banks and forms of helicopter money. Even the Minskian 'employment of last resort' proposal has been resurrected (Saez and Zucman, 2020). The only certainty in a time of massive uncertainty is that this pandemic is and will continue to dramatically affect the labour market and the economy on a global scale.

The direct and indirect impacts of the pandemic are evident in many realms of the economy, from the organisation of production and global value chains, to patent systems and appropriability conditions in the pharmaceutical sector, to the provision of health as a public good, to

* The authors acknowledge support from European Union's Horizon 2020 research and innovation programme under grant agreement No. 822781 GROWINPRO – Growth Welfare Innovation Productivity.

1 The International Monetary Fund's April 2020 World Economic Outlook projects a drop of 3% for the world economy. During the 2009 financial crisis, the drop was 0.1%.

**Armanda Cetrulo**, Scuola Superiore Sant'Anna, Pisa, Italy.

**Maria Enrica Virgillito**, Scuola Superiore Sant'Anna, Pisa, Italy.

the study of unconventional fiscal and monetary policies (Lucchese and Pianta, 2020). Additionally, the implications for the organisation of the workplace and the work process are going to be significant. Social distancing is expected to jeopardise business and employment opportunities in a labour market that is defined by strong inequalities and precarious jobs.

Doing 'smart work' implies access to both material and immaterial infrastructure in order to enable the actual implementation of remote working, e.g. a computer at home, access to the Internet and working tasks and activities that can be executed from home (e.g. imputing, elaborating and publishing data and information). However, a large fraction of European workers do not meet these feasibility conditions. Analysis even for the US, which is supposed to be more digitalised, indicates a *digital divide*; in particular, access to a high-speed Internet connection is highly correlated with income, and in turn the possibility to work from home increases with income level (Chiou and Tucker, 2020).

Italy was the first European country to implement general social distancing policies. In the second half of March, a Decree Law from the Prime Minister formally closed the so-called 'non-essential' working activities and once again recommended the application of smart work even for private companies, following a disposition at the end of February that strongly encouraged the implementation of 'agile' work for the public administration. The Italian press and public opinion have welcomed social distancing as a chance to actually implement smart working on a larger scale given that in 2018 only 3.6% of workers regularly worked from home (Eurostat, 2020). Given the lockdown and the requirements of social distancing, working from home seems to be a very sensible choice. It would *in principle* allow for the provision of information-based services, which in the 'smart era' is supposed to be the majority of the labour force.

But, how many workers are actually in a situation to perform smart work? And what are the underlying characteristics in terms of contractual categories and wage distributions? This article looks at the Italian occupational structure, quantifies the jobs that can be done at home and defines the composition of the underlying labour force in terms of occupational, wage and contractual distributions. To address these issues, we analyse the Italian dictionary of occupations *Indagine Campionaria delle Professioni* (ICP), a survey at the five-digit level structured like the US O*NET dataset and linked with the Italian Labour Force Survey (ILFS). Our analysis reveals a series of alarming results. First, only 30% of all of the occupations in the survey can be performed from home, accounting for 6.7 million workers. The remaining nearly 16 million workers perform tasks that they cannot do remotely. According to our second finding, the occupations that may work from home are extremely concentrated among managerial and executive categories, academics, technical professionals and clerical support workers. Sales and service workers, manual operators, artisans and elementary occupations have little to no opportunity to work from home. Occupational inequality translates into inequalities in wage distribution. Indeed, those who can work from home are paid substantially more. Finally, looking at the contractual framework (whether permanent, autonomous, temporary) we note that temporary workers are mostly concentrated in occupations that cannot be performed at home.

## Data, methodology and results

The following results adapt and expand the methodology proposed by Dingel and Neiman (2020), who analyse the occupations that can be carried out from home in the US starting from the O*NET dictionary of occupations. The analysis for Italy is based on an integrated database that includes the ICP and the Italian National Institute of Statistics (Istat) Labour Force survey, updated in 2016.

From the ICP dataset we are able to derive information regarding generalised work activities and the working context of five-digit occupations. The ICP – a survey conducted by the National Institute for Public Policy Analysis (INAPP) in collaboration with Istat – represents the only European source comparable with the American O*Net database, the latter being the most comprehensive database reporting qualitative and quantitative information on tasks, skills, work contexts and organisational characteristics at the five-digit level of observation. The construction of the dataset entails a complex, multi-layer strategy of data collection and information processing allowing for both detailed occupational descriptions and inter-occupational comparability (more in Cetrulo et al., 2019).

Currently, two waves of the ICP database are available (2007 and 2012) with a spectrum covering 797 occupational codes, excluding the armed forces. We rely on the 2012 wave. The interviews were administered to 16,000 Italian workers to ensure statistical representation with respect to sectorial, occupational, dimensional and geographical heterogeneities. Both O*NET and ICP questions are organised in six main sections, expressions of a content model that simultaneously provides information from both a job-oriented and a worker-oriented perspective. The descriptors are: worker characteristics (enduring abilities and work style of workers), worker requirements (skills and education), occupational requirements (organisational and work context), experience requirements (training, cross functional skills), workforce characteristics (labour market information) and occupation-specific information (generalised activities and work context). In so doing, descriptors are formulated by making it possible to distinguish, for instance, inner individual abilities from competences acquired on the job. For each question, two rating scales are generally provided: level and importance.

To identify the activities that cannot be carried out from home, a series of questions belonging to the generalised activities and work context sections of the ICP have been selected.

These questions provide insight on the relative importance of:

- performing activities involving (i) use, control and repairing of machines, equipment, vehicles,[2] (ii) social contact, taking care of or assisting others,[3] (iii) email correspondence;
- performing activities which (i) are carried out outdoors, (ii) require exposure to diseases and infections, (iii) imply the execution of risky movements or the wearing of protective equipment.[4]

For each five-digit occupation, these variables are ranked according to an importance scale ranging from 0 to 100. Professions classified as 'not from home' consisted mostly of respondents who spend a large fraction of their working time in external environments or use equipment, machinery or tools or had continuous contact with the public. For example, if for a given occupation, most respondents report that it is very important to control machinery and use equipment, this occupation cannot be carried out from home. Similarly, if most of the respondents report that they perform outdoor tasks for most of their working time, this occupation cannot be carried out from home. Additionally, if sending emails is a very infrequent activity for a given occupation, the occupation cannot be performed from home. The classification is useful in order to identify jobs that can and cannot be executed from home on the basis of the actual performed tasks and work contexts and starts by excluding all those occupations that require working in a well-defined physical space (e.g. because of the use of working instruments or because of intensive social contact).[5]

After identifying the occupational categories at the four-digit level, these are aggregated at the one-digit level

Table 1
**Top ten occupations that can and cannot be performed from home, ISCO classification**

| | |
|---|---|
| **Cannot be performed at home** | |
| 644 | Specialised forestry workers |
| 711 | Plant and machinery operators for the extraction and initial treatment of minerals |
| 724 | Machinery workers in plants for the mass production of wooden items |
| 743 | Agricultural machinery drivers |
| 841 | Unqualified mining and quarrying personnel |
| 842 | Unqualified construction personnel and similar professions |
| 716 | Plant operators for the production of thermal energy and steam, for waste recovery and for the treatment and distribution of water |
| 645 | Fishermen and hunters |
| 712 | Metal processing and hot working plant operators |
| 612 | Craftsmen and skilled workers in the construction and maintenance of building structures |
| **Can be performed at home** | |
| 252 | Specialists in legal science |
| 431 | Employees in charge of the administrative management of logistics |
| 254 | Specialists in linguistic, literary and documentary disciplines |
| 411 | Secretarial and general affairs employees |
| 121 | Entrepreneurs and directors of large companies |
| 122 | Directors and general managers of companies |
| 211 | Specialists in mathematical, computer, chemical, physical and natural sciences |
| 331 | Technicians of the organization and administration of production activities |
| 432 | Economic, accounting and financial management employees |
| 251 | Management, commercial and banking science specialists |

Source: ICP-ILFS, 2016.

2 For example, "Handling and moving objects", "Managing machines and processes", "Manoeuvring vehicles, vehicles and equipment", "Repairing and maintaining mechanical equipment".
3 "Working in direct contact with the public and performing", "Assisting and taking care of others".
4 "How often your occupation requires you to work outdoors exposed to all climatic conditions", "How often are you exposed to contaminants", "How often does your work require exposure to dangerous equipment?".
5 In case of the application of social distancing, an occupation as primary school teacher, which could not be carried out from home according to our classification, will eventually be performed from home. In fact, there are tasks, largely related to activities as "taking care of others" or "working with the public" that could potentially be digitalised, however, at the cost of entirely reconfiguring the very nature of the profession.

Figure 1

**Percentage of employees in occupations that can and cannot be performed from home**

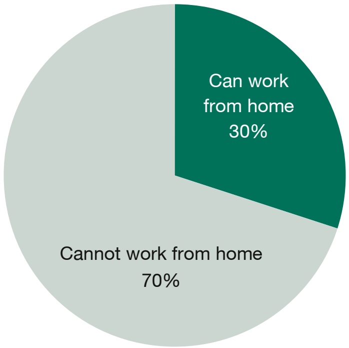

Source: ICP-ILFS, 2016.

Figure 2

**Distribution of occupations that can and cannot be performed from home**

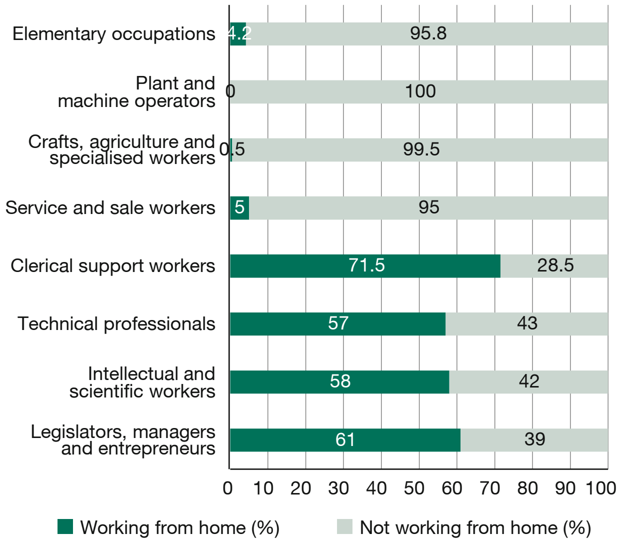

Note: One-digit level, ISCO classification.

Source: ICP-ILFS, 2016.

according to the ISCO classification and are then linked to the Labour Force Survey providing information on the number of employees, wages, contractual types and socio-demographic characteristics of workers (age, gender and level of education).

Table 1 presents ten occupations which can be performed at home and ten which cannot at the three-digit level. The occupations are ranked in terms of the number of variable co-occurrences, out of thirty selected variables from the ICP, which define an occupation as unfeasible from home. The higher the number of co-occurrences, the higher the ranking of the occupation.

Occupations like woodcutters, miners, construction workers and fishermen rank among the top professions that cannot be performed at home. On the contrary, occupations involving specialised field knowledge, such as legal or linguistic experts, as well as managerial and executive posts are among the top jobs that can be performed remotely. In terms of organisational hierarchies, occupations that cannot be performed remotely tend to be located at the lower end of the employment structure. Those who self-organise their work activity, give orders or are responsible for high-level administrative activities can operate remotely.

Figure 1 shows the corresponding number of employees for each of the two categories. Only 30% of the workforce, or 6.7 million workers, have an occupation that can be done from home. For the remaining nearly 16 million workers, the work tasks and context do not make working from home feasible. This figure is in line with Dingel and Neiman's (2020) finding that 37% of occupations can be conducted from home in the US. This share is probably an overestimate since the classification arises from the identification of jobs that cannot be done from home, but does not ensure that those who can work from home are actually doing so. Another overestimation bias derives from the complementarity between occupations and from the integration of work processes: take the case of travel agencies, whose work, although remotely executable, is simply not done because of the lack of clients for hotels and restaurants.

By aggregating at the one-digit level according to the ISCO classification, it is possible to analyse the distribution of the occupations that can or cannot be carried out from home within each of the eight professional macro groups. In Figure 2, a highly polarised occupational structure emerges with a strong concentration of opportunities to work from home for the first four occupational groups. Agile work is feasible for 60% of those who are at the top of the organisational hierarchy (managers, entrepreneurs and legislators), for scientific-academic professions and for technical professionals. It increases to 70% for administrative tasks. For the lower part of the distribution, the scenario radically changes. For service occupations, such as entertainment operators, service and sales workers, artisans, plant and machine operators as well as elementary professions, the opportunities for remote working reduce drastically with variations ranging from 5% to 0%.

Figure 3

**Median monthly wage distribution of occupations that can and cannot be performed from home**

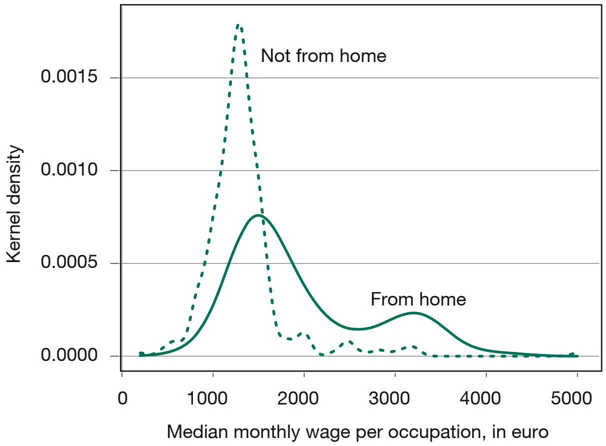

Source: ICP-ILFS, 2016.

Figure 4

**Contractual framework of occupations that can and cannot be performed from home**

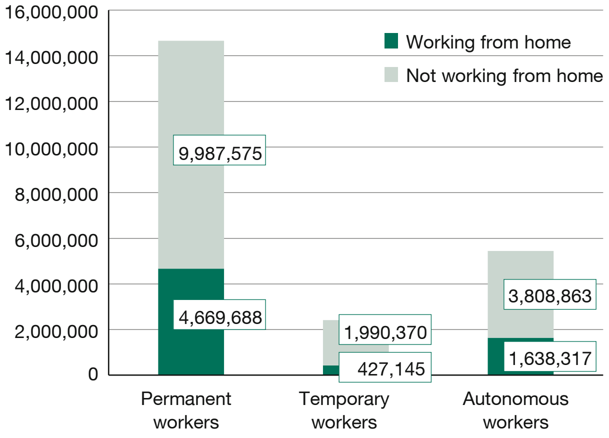

Source: ICP-ILFS, 2016.

Inequality in occupations clearly maps into inequality in the corresponding wage structure. In fact, while the distribution of median wages for not-working from home occupations is largely concentrated in the range of €500-1,800 per month, working from home occupations record median wages largely concentrated in the range of €1,000-2,000 per month (see Figure 3), with a fatter right tail.

The nearly 16 million workers who do not have the chance to work from home not only earn less money, but they are also among those most exposed to closer social proximity and the possibility of getting infections, the latter being among the dimensions covered by our classification of working/not working from home (Barbieri et al., 2020). This polarisation in the wage structure is also reflected in the contractual structure which sees temporary workers largely concentrated in professions that cannot be carried out from home, affecting almost two million workers, to which 3.8 million self-employed workers should be added to encompass all at-risk jobs, as shown in Figure 4. These are workers who earn less yet face the greatest risk of unemployment due to their contractual arrangement and have the most difficulty executing their work remotely.

## Labour market inequalities during the coronavirus pandemic

Overall, the spreading of the pandemic exacerbates a series of existing inequalities and increasing vulnerabilities. Although the common perception is that the pandemic is 'the great equaliser', workers' tasks, contractual framework and position in the internal organisational hierarchy strongly affect their ability to work remotely. Take two archetypical cases: a manager and a manual worker. While the first has the chance to keep giving orders from home, the second can hardly drive a vehicle or use an instrument remotely. Additionally, while the first is relatively less exposed to infections, contagious diseases and work accidents, the opposite is true for the second. This means that the coupling of the pandemic and social distancing are conflating diverse risks: *health risks* (exposure to social contacts is higher for low income occupations), *income risks* (exposure to job losses is higher for temporary, low income occupations), and *employment risks* (feasibility of remote work is lower for low income occupations).

Whereas those who are unable to work remotely are exposed significantly more to risks right from the outset, the risk exposure of those working from home are heterogeneous as well. Take the case of two clerical workers: one is employed by a small enterprise and the other by a big one (i.e. more than 50 employees). Although performing quite similar job tasks, and therefore both classified as occupations which can be done from home, the first worker is going to face more difficulties and less support in setting up a work station from home vis-à-vis the second due to the fact that small firms are generally less capable of digitalising their administrative activities.

If workers are not equal when facing the consequences of the pandemic, policies should support forms of internal reorganisation of the workplace and encourage safe conditions. This means that together with the provisions of safety equipment (still not guaranteed), a more comprehensive intervention should seek to reduce working hours (at constant wages) and rearrange shifts.

The reduction of working hours at constant wages would be a much more equitable scheme (with potentially positive feedback on the demand side) rather than redundancy payment schemes and supposedly generalised lockdowns. A general organizational flexibility with reduced working hours at the same wage would allow for the maintenance of productive capacity, job and income guarantees.

All of this, however, requires a massive reorganisational effort of the production process and clashes with the lack of organisational capabilities of Italian firms, the majority of which employ less than ten employees. As largely documented (Dosi et al., 2019), Italian firms are strongly heterogeneous in performance variables and underlying organisational capabilities. Additionally, they tend to display a poor attitude towards workplace collaborative practices and job rotation schemes, which might be very helpful in times of flexible rostering, and look to be abundantly equipped by a rigid and hierarchical internal division of labour (Cetrulo et al., 2019).

To mitigate these structural divergences, policies play a crucial role. First, firms should be monitored for the provision of safety devices. Second, a nationwide plan to provide equal opportunities for workers who are (technically) capable of performing smart work should be put in place. Finally, working shifts should be reorganised and working hours should be reduced. Taken together, these initiatives offer a viable solution.
